# Continuous and selective measurement of oxytocin and vasopressin using boron-doped diamond electrodes

**DOI:** 10.1038/srep32429

**Published:** 2016-09-07

**Authors:** Kai Asai, Tribidasari A. Ivandini, Yasuaki Einaga

**Affiliations:** 1 Department of Chemistry, Faculty of Science and Technology, Keio University, 3-14-1 Hiyoshi, Yokohama 223-8522, Japan; 2 Department of Chemistry, Faculty of Mathematics and Science, University of Indonesia, Kampus UI Depok, Jakarta 116424, Indonesia; 3 JST-ACCEL, 3-14-1 Hiyoshi, Yokohama 223-8522, Japan

## Abstract

The electrochemical detection of oxytocin using boron-doped diamond (BDD) electrodes was studied. Cyclic voltammetry of oxytocin in a phosphate buffer solution exhibits an oxidation peak at +0.7 V (*vs*. Ag/AgCl), which is attributable to oxidation of the phenolic group in the tyrosyl moiety. Furthermore, the linearity of the current peaks obtained in flow injection analysis (FIA) using BDD microelectrodes over the oxytocin concentration range from 0.1 to 10.0 μM with a detection limit of 50 nM (*S*/*N* = 3) was high (*R*^2^ = 0.995). Although the voltammograms of oxytocin and vasopressin observed with an as-deposited BDD electrode, as well as with a cathodically-reduced BDD electrode, were similar, a clear distinction was observed with anodically-oxidized BDD electrodes due to the attractive interaction between vasopressin and the oxidized BDD surface. By means of this distinction, selective measurements using chronoamperometry combined with flow injection analysis at an optimized potential were demonstrated, indicating the possibility of making selective *in situ* or *in vivo* measurements of oxytocin.

Oxytocin, which is known as a simple cuddle chemical, is a nonapeptide with many important biological functions. In addition to its functions as a hormone in facilitating uterine contractions and mammalian milk ejection, it was also discovered that oxytocin works as a neurotransmitter and is considered to mediate social behavior, such as pair bonding and instinctive maternal aggression^1^,^2^,^3^. Several positive effects on a range of social behavior have led to a high level of interest in oxytocin as a support treatment for a number of diseases, including autism, depression, anxiety, and schizophrenia. Such treatment depends greatly on the dose level as generally a small dose is ineffective, while an excessive dose can give results opposite to those intended^4^,^5^,^6^.

In addition, an understanding of the fundamental science underlying the mechanism for the function of oxytocin in the brain is urgently needed in order to comprehend the substantive function of oxytocin^7^. Hence, making oxytocin measurements in the brain is a very important issue. In particular, real-time *in vivo* measurements of oxytocin are required in order to observe the secretion of oxytocin with a millisecond time resolution, as well as the actual concentration of secreted oxytocin, which may help reveal the relationship between oxytocin and its physiological effect. However, making reliable oxytocin measurements in the brain is considered to be very difficult. Therefore, it is still not clear when, where, and how much oxytocin is released, and what its fundamental role is in the brain.

On the other hand, in the region where oxytocin is secreted in the hypothalamus, another peptide, vasopressin, is also secreted. Vasopressin is also a nonapeptide with a quite similar structure to oxytocin. The amino acid sequences of oxytocin and vasopressin, respectively, are as follows:

Oxytocin: Cys-Tyr-**IIe**-Gln-Asn-Cys-Pro-**Leu**-Gly-NH_2_

Vasopressin: Cys-Tyr-**Phe**-Gln-Asn-Cys-Pro-**Arg**-Gly-NH_2_

The difference between these two peptides is only in two amino acids (highlighted in bold). Inside each peptide residue, two cysteine molecules form a disulfide bond.

Due to the similarity of the structures, both peptides compete for antibodies that may have some degree of affinity for peptides with similar amino acid sequences leading to low specificity in the main available method for oxytocin detection, i.e. radio immunoassay (RIA)^7^,^8^,^9^. The low specificity problem can be solved using mass spectrometry combined with liquid chromatography^7^. However, it takes at least some minutes to make one measurement due to microdialysis of the sample or the retention time in liquid chromatography. A technique for making real-time measurements is one of the requirements that will enable fundamental studies of oxytocin to be made. Thus, the use of electrochemical methods, which respond within the order of milliseconds, is a promising way forward.

Furthermore, of the 20 standard proteinogenic amino acids, only cysteine, tryptophan, and tyrosine are known to be electrochemically active at moderate potential^10^,^11^,^12^,^13^. Therefore, peptides or proteins that contain at least one of these three amino acids in their structure can, theoretically, be detected electrochemically. However, only a few reports have been published in regard to the direct electrochemical detection of peptides or proteins^10^,^11^,^14^,^15^, which might be due to the lower sensitivity of electrochemical methods compared to those of immunoassay or mass spectrometry. In addition, passivation of the electrode surface due to adsorption of oxidized or polymerized protein molecules causes low reproducibility of the measurements^16^.

One promising electrode material is boron-doped diamond (BDD), which can be used to overcome these difficulties. It is widely known that BDD electrodes have low background current and capacitance, giving the superiority for potential sweep analyses^17^. BDD also has high chemical and physical stability, which helps maintain the reproducibility of the signal^17^,^18^,^19^. Furthermore, due to its wide potential window for aqueous solution, a wide range of substances can be detected using BDD electrodes^20^,^21^,^22^,^23^,^24^. BDD also has high resistance to fouling because of the carbon sp^3^ structure of the sample-sensor interface^25^,^26^, which is advantageous for *in vivo* measurement. Moreover, the termination at the BDD surface can be electrochemically altered from reduced to oxidized, and *vice versa*^27^,^28^,^29^,^30^. Selective *in vitro* and *in vivo* measurements of dopamine^30^,^31^,^32^,^33^, disulfide-containing proteins^34^,^35^, as well as adenosine phosphates^36^, using the phenomena have been reported. These anodic and/or cathodic treatments of BDD surfaces can be used as pre-treatments as well in order to reproduce the electrochemical signals collected at BDD electrodes, removing or reducing the substances adsorbed on the surface during measurements^22^,^25^,^37^. Especially, for *in vivo* measurements, BDD microelectrodes have been fabricated for dopamine detection in the brain^32^, glutathione detection in tissue^35^, and pH monitoring in the stomach^38^.

In the present work, first, electrochemical detection of oxytocin and vasopressin was studied using BDD electrodes. The oxidation peaks of oxytocin and vasopressin could clearly be distinguished due to the difference in electrostatic interactions between the peptides and BDD surface. By means of this distinction, selective measurements using chronoamperometry combined with flow injection analysis at an optimized potential were successfully demonstrated using BDD microelectrodes. The ability to alter the BDD surface from a reduced to an oxidized form, and *vice versa*, makes a major contribution to the selectivity, which would lead to selective *in vivo* measurements of oxytocin.

## Results and Discussion

### Electrochemistry of oxytocin

**Figure 1:**
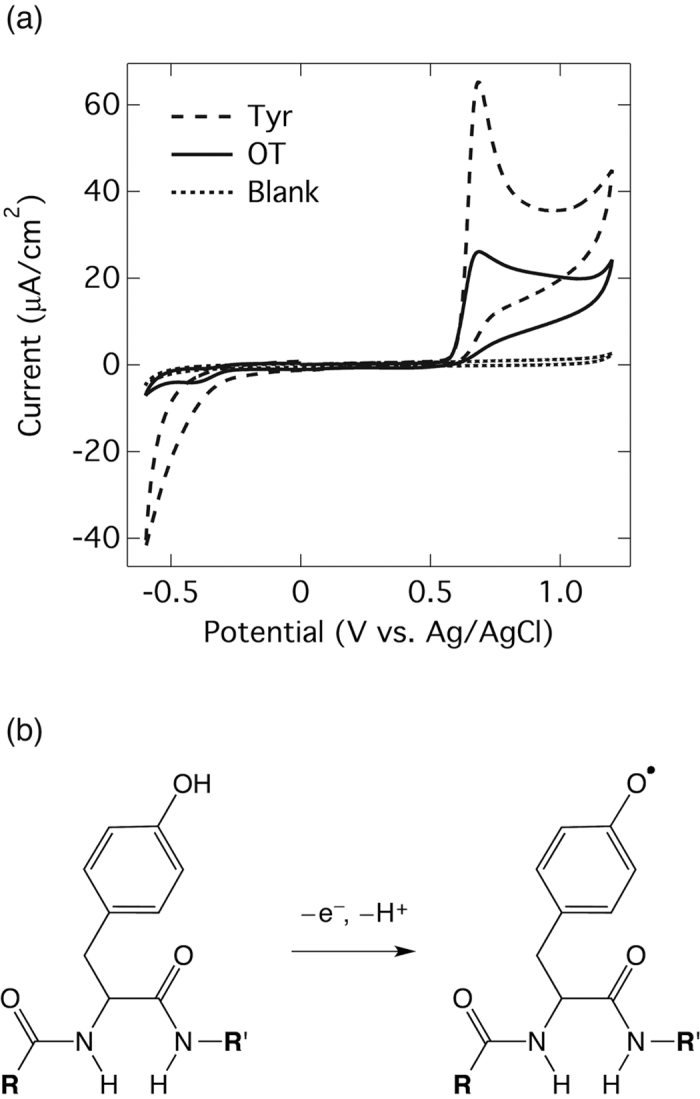
The study of electrochemical behavior of oxytocin by cyclic voltammetry. (**a**) Cyclic voltammograms of PBS (0.1 M, pH 7.4) using as deposited-BDD electrodes in the absence (Blank) and the presence of 0.1 mM oxytocin (OT) and 0.1 mM tyrosine (Tyr). The scan rate is 100 mV/s and starts from 0.0 V (positive scan). (**b**) The proposed oxidation mechanism of oxytocin at the tyrosyl moiety. **R** and **R**’ represent the remaining parts of the oxytocin.

The electrochemical behavior of oxytocin was studied using cyclic voltammetry. Figure 1(a) shows cyclic voltammograms (CVs) using as-deposited BDD electrodes for 0.1 M phosphate buffer solution (PBS, pH 7.4) in the absence and the presence of 0.1 mM oxytocin. A scan rate of 100 mV/s was applied. Whereas no peak appears in the absence of oxytocin, a well-defined oxidation peak in the presence of oxytocin can be seen at +0.7 V (*vs*. Ag/AgCl). When cyclic voltammetry was applied over a wider potential range, in addition to the peak at +0.7 V, a wide and broad oxidation signal appeared at around +1.5 V (see Supplementary Information Fig. S1). This signal was broad due to overlapping with the on-set of oxygen evolution reaction. It has been reported that disulfide bonds in peptides or proteins can be oxidized above +1.5 V at the surface of a BDD electrode^34^,^35^. Comparison of the CVs of oxytocin and cystine, which has a disulfide bond, confirmed that the broad peak at around +1.5 V can be attributed to oxidation of the disulfide bond. However, considering the oxidation peak at +0.7 V is clearer and provides a higher signal at lower potential than that at +1.5 V, the peak at +0.7 V was selected for the next study of oxytocin.

Since, of the nine amino acids contained in oxytocin, only tyrosine can be electrochemically oxidized, cyclic voltammograms of tyrosine were also done for confirmation. Comparison between the CVs of tyrosine and oxytocin (Fig. 1a) show that they are similar, with a typical oxidation potential at +0.7 V. Taking this similarity into account, it can be said that oxidation of oxytocin occurs at the tyrosine moiety.

The difference in peak current might mostly depend on the diffusion coefficient. Assuming a diffusion controlled electrochemical reaction, peak current in CV is proportional to the square root of diffusion coefficient (*D*). *D* is inversely proportional to the radius of molecule (*r*_0_) under the prediction of *D* by Stokes-Einstein equation. *r*_0_ of oxytocin (*ca*. 10.5 Å ref. 39) is about 2.3-fold larger than that of tyrosine (*ca*. 4.5 Å). Therefore, the peak current of tyrosine should become 1.5-fold higher than that of oxytocin. Although the difference in peak current in this experiment was larger than that (*ca*. 2.5-fold), the dominant factor for the different peak currents might be the diffusion coefficient.

The electrochemistry of tyrosine has been studied using electrodes made of several types of material, including BDD^13^,^40^,^41^. The general understanding is that the oxidation initially occurs at the hydroxyl group of the phenol part with the removal of 1 electron and 1 proton, leading to the formation of the radical shown in Fig. 1b. After that primary single electron oxidation, further oxidation or chemical reactions such as polymerization lead to a complex mixture of substances. Furthermore, once the tyrosine in the peptide is oxidized, it can induce cleavage of the peptide backbone, especially cleavage at the C-terminal of the tyrosine residue^41^.

It is reported that tyrosine (p*K*_a_ (at phenol) = 10.0, Table 1) oxidation at BDD electrode is pH dependent with a slope of (dE/dpH) *ca*. 60 mV/pH up to pH 9.8 with the increase in pH, above which it becomes pH-independent^40^. This well corresponds to the oxidation mechanism of 1 electron and 1 proton reaction. Oxidation of oxytocin would also show similar pH dependency because the p*K*_a_ of phenolic group in oxytocin (p*K*_a_ = 10.1) is almost the same as that of tyrosine.

Besides the oxidation peak, a reduction peak at around −0.4 V also appeared (Fig. 1a). However, this peak was not observed when the voltammogram scan was done in the opposite direction (see Supplementary Information Fig. S1), indicating that this peak was generated from substances produced as a result of oxytocin oxidation. When more scans were cycled (more than 50 times), the oxidation signal coupled to the reduction appeared at −0.3 V and the signals of the redox couple became larger. This redox reaction would correspond to the reaction of quinoic strucure^42^,^43^ that would be one of the products of oxytocin oxidation^41^.

**Figure 2:**
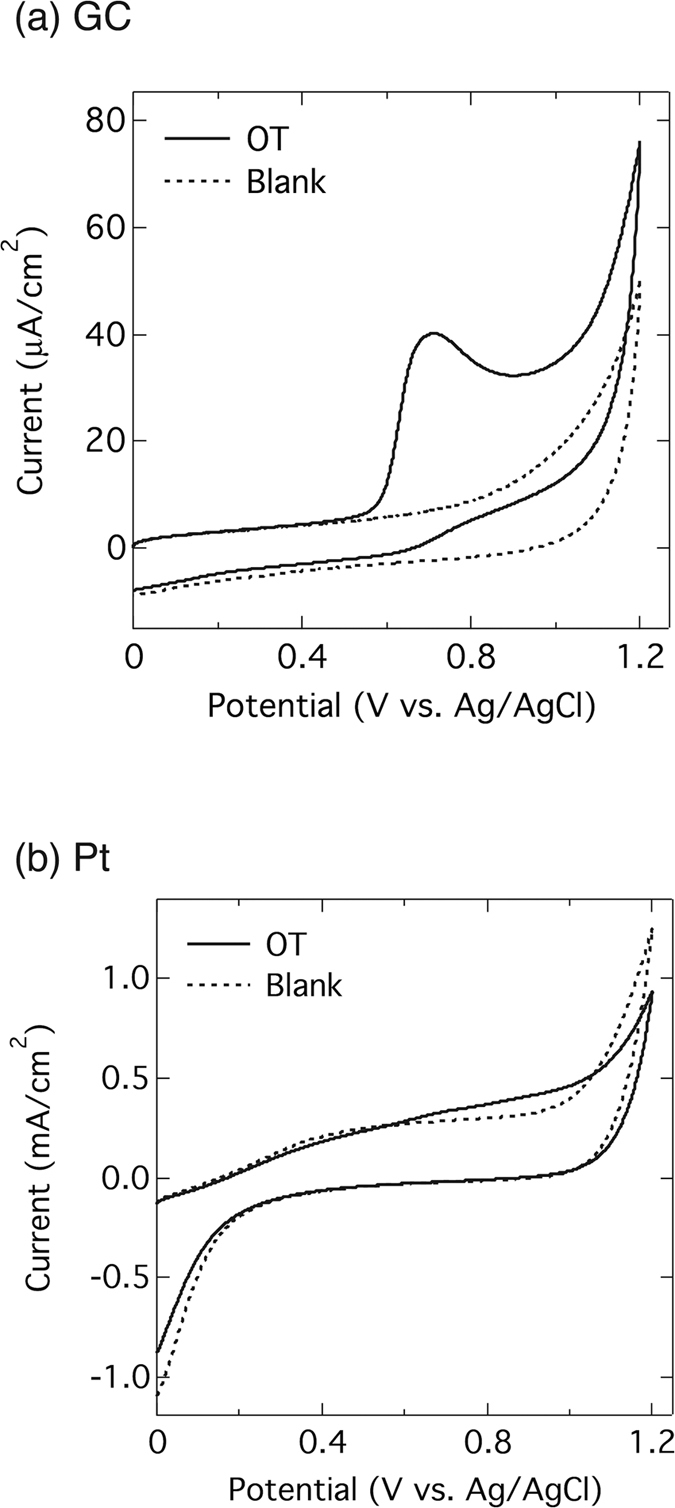
Electrochemical behavior of oxytocin on other electrodes. Cyclic voltammograms of PBS (0.1 M, pH 7.4) with GC (**a**) and Pt (**b**) electrodes in the absence (dashed line) and the presence (solid line) of 0.1 mM oxytocin. The other conditions are as in Fig. 1(a).

Comparisons were conducted using platinum (Pt) and glassy carbon (GC) electrodes. Whereas no peak was observed in the CV with the Pt electrode (Fig. 2b), a well-defined oxidation peak appeared with the GC electrode (Fig. 2a). The peak potential as well as the peak current when using the GC electrode is almost the same as that with the BDD electrode since both the GC and BDD electrodes are carbon-based, therefore, similar oxidation mechanisms are expected to occur. However, the voltammetric signal of oxytocin is much broader with the GC electrode. Furthermore, in the absence of oxytocin, the GC electrode has higher background current. Therefore, when the oxidation peak current for oxytocin (signal) is compared to the current at the same potential in the absence of oxytocin (background), the signal to background ratio (*S*/*B*) with the BDD electrode (*S*/*B* = 21.0) is about 4 times higher than that with the GC electrode (*S*/*B* = 5.0), showing the BDD electrodes to be superior in terms of this voltammetric analysis. In the case of the Pt electrode, there is an increase in current in the potential range from +0.5 to +0.8 V due to the presence of oxytocin. However the high background current in the voltammograms makes it difficult to identify any oxidation peak.

### Surface transformation and selective measurement

**Figure 3:**
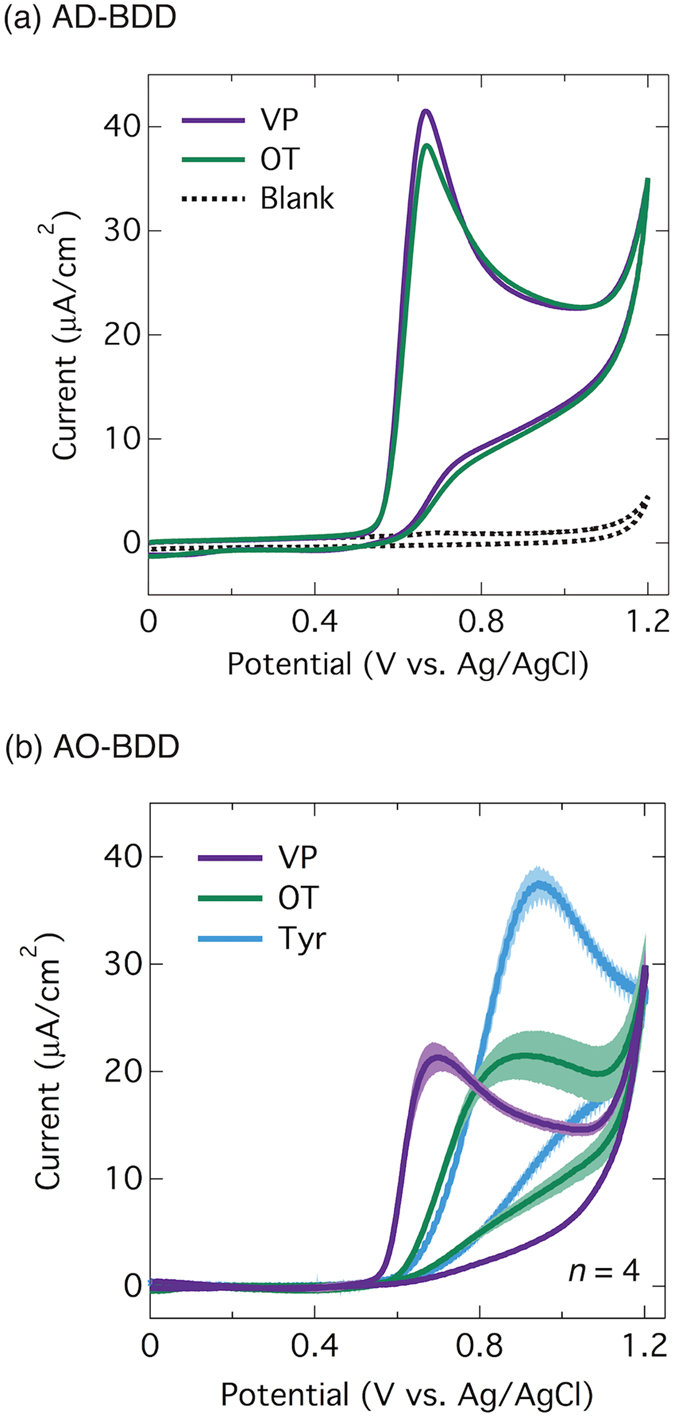
Comparison between the CVs of oxytocin and vasopressin on AD-BDD and AO-BDD. (**a**) Cyclic voltammograms of 0.1 mM oxytocin (OT) and vasopressin (VP) in PBS (0.1 M, pH 7.4) using as-deposited BDD electrodes. (**b**) Cyclic voltammogram of PBS (0.1 M, pH 7.4) using an anodically oxidized BDD electrode in comparison with voltammograms in the presence of 0.1 mM oxytocin (OT), vasopressin (VP) and tyrosine (Try). The other conditions are similar to those in Fig. 1(a).

In preparation for measurements in biological samples, the selectivity between oxytocin and vasopressin was investigated. Figure 3a shows a comparison between the CVs of vasopressin and oxytocin on as-deposited BDD (AD-BDD). Identical oxidation peaks can be seen at the same potential (+0.7 V), which is reasonable since the electrochemically active site of vasopressin is also the tyrosine moiety. Although cathodic reduction recovered the deteriorated sensitivity of BDD electrode after several measurements as previously reported^36^, those cathodically-reduced BDD (CR-BDD) showed the same voltammogram as AD-BDD.

The BDD electrodes were then anodically oxidized. AD-BDD is known to have mainly hydrogen termination since it is prepared in a hydrogen plasma^44^. It is also reported that anodic oxidation of BDD electrode can convert some of these hydrogen functional groups into oxygen functional groups^27^,^28^. Anodic oxidation of as-deposited BDD can increase the oxygen to carbon (O/C) ratio from 0.02 up to 0.3^27^,^28^. Characterization of such anodically-oxidized BDD (AO-BDD) surface by XPS shows that it contains many C-O functional groups^27^,^30^,^45^,^46^,^47^. A surface rich in C-O functionalities is believed to be in an electrostatically negative state due to the relatively high electronegativity of the oxygen atoms or just due to the negative charge of C-O functionalities such as carboxyl groups. In fact, a number of reports support the changes observed in the electrostatic interactions between AD-BDD and AO-BDD^28^,^31^,^36^,^46^.

Figure 3b shows the average CVs (*n* = 4) of oxytocin and vasopressin at an AO-BDD electrode. The figure clearly shows the oxidation peaks of oxytocin and vasopressin to be at different potentials. Whereas the oxidation peak of vasopressin at +0.7 V hasn’t changed, the oxidation peak of oxytocin has shifted to a more positive potential (from +0.7 V at AD-BDD to +0.9 V at AO-BDD). In addition, comparison with the voltammogram of tyrosine itself shows the oxidation peak for this has shifted to an even more positive potential (*ca*. +0.95 V).

Similar to oxytocin, the electrochemical active site of vasopressin is also tyrosine. Therefore, the similar oxidation potentials of tyrosine and oxytocin suggest that there is a shift in oxidation potential due to vasopressin and not one due to oxytocin. It seems that a stronger interaction between vasopressin and the electrode surface occurs at the AO-BDD surface compared to the AD-BDD surface. One possible explanation for this result is the electrostatic interaction between the electrode surface and the peptides. As previously mentioned, the presence of oxygen sites causes the surface of the BDD electrode to be relatively negative^19^. On the other hand, some proteins are charged due to the composition of their amino acids. The charges are reflected in their isoelectric points (pI). Table 1 shows the pIs of the peptides and some related amino acids that contribute to the different compositions of oxytocin and vasopressin^48^,^49^. The table shows that the pI of vasopressin (10.9) is much higher than those of oxytocin and tyrosine (7.7 and 5.7 respectively). With a pI of 10.9 in pH 7, which was the electrolyte condition for the CV measurements, vasopressin has positive charge, while oxytocin is neutral. As a result, the electrostatically negative surface of the electrode attracts vasopressin molecules and causes the lower oxidation potential of vasopressin. Furthermore, since leucine, isoleucine, and phenylalanine have similar pIs (between 5.5 and 6.0.), the arginine moiety (pI = 11.2) in the vasopressin structure is considered to be the site most responsible for the interaction between vasopressin and the electrode surface.

The level of electrostatic interaction generally depends on the ionic strength. The ionic strength of the 0.1 M PBS (pH 7.4) is 262 mM, which is higher than physiological ionic strength (100 ~ 200 mM). The same measurement was conducted under near-physiological condition. Tris buffer (ionic strength: 178 mM) was chosen as buffer saline and AO-BDD microelectrode was used. Still the difference in oxidation potential between oxytocin and vasopressin was observed (see Supplementary Information Fig. S3). This result shows that the selective detection of oxytocin and vasopressin on AO-BDD can be applied to *in vivo* measurements.

Note that a pre-treatment consisting of cathodic reduction (−3.0 V for 5 min) and then anodic oxidation (+3.0 V for 20 min) was conducted before and between each measurement for making oxygen-terminated surface reproducibly. Neither reproducible voltammograms nor comparison of the electrochemical behavior could be obtained without this pre-treatment because the adsorption of oxidized molecules caused the signal to be degraded. Especially cathodic reduction would play an important role in the signal recovery. This kind of strong and repeated electrochemical treatment, which is necessary and effective for making selective and reproducible measurements using BDD electrodes, is difficult to apply to other types of electrode.

### Measurements using BDD microelectrodes: toward *in vivo* measurements

**Figure 4:**
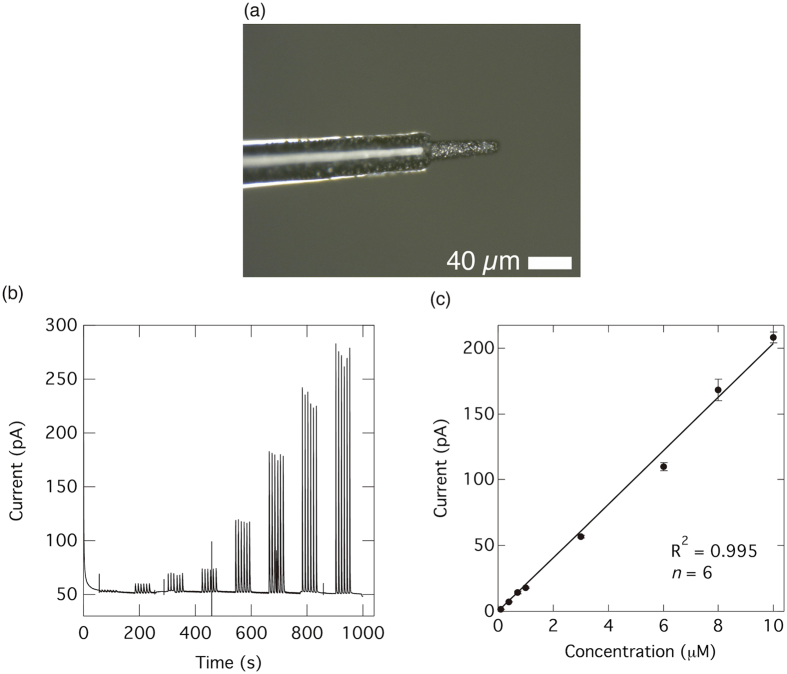
Flow injection analysis for oxytocin on BDD microelectrode using chronoamperometry. (**a**) Optical microscopy image of a BDD microelectrode. (**b**) Flow injection analysis of oxytocin in a Tris buffer (pH 7.4) using a BDD microelectrode. The applied potential was 1.0 V. Oxytocin concentrations of 0.1–10 μM were injected 6 times for each concentration. (**c**) Calibration curve extracted from the current recorded in (**b**) against the oxytocin concentration (*n* = 6). Error bar shows standard deviation.

Aiming at *in vivo* measurements, BDD microelectrodes are used since a spatial resolution of the order of a micrometer is required. Flow injection analysis (FIA) of oxytocin using an as-deposited BDD microelectrode was conducted for monitoring the concentration change as a calibration for *in vivo* measurement. Chronoamperometry was selected as the detection technique. Initially, the limit of detection (LOD) of oxytocin was calculated by preparing a calibration curve. Standard oxytocin solutions with a concentration range from 0.1 to 10 μM were injected and the signal currents generated at an applied potential of +1.0 V using a BDD microelectrode (Fig. 4a) were recorded. Tris (pH 7.4) was used as the buffer solution. Figure 4b displays the signal currents, which were observed after injection of the oxytocin samples. The relationship between the signal current and the oxytocin concentration is plotted in Fig. 4c, which shows the correlation to be highly linear (*R*^2^ = 0.995) with an LOD of 50 nM (*S*/*N* = 3). These results demonstrate the possibility of performing real-time detection of oxytocin in the nM concentration range.

This measurement, however, is not selective for oxytocin. When vasopressin was added to the oxytocin solution, the current increased as both peptides are oxidized at the same potential. Therefore, a further investigation using an anodically-oxidized BDD microelectrode was conducted. As shown in Fig. 3b oxidation of vasopressin starts at a potential of about +0.5 V, while that of oxytocin starts around +0.7 V. Thus, it is expected that selective measurements of vasopressin can be done at applied potentials below +0.7 V, while at applied potentials higher than +0.7 V both vasopressin and oxytocin will be measured.

Prior to use, the electrode surface was anodically oxidized at +3.0 V as also conducted for the CVs in Fig. 3. Flow injection analysis at +0.6 V for 5 consecutive sample injections was performed. However, in contrast to the results using non-oxidized BDD electrodes, when standard solutions of vasopressin were injected, the signal currents kept decreasing. The decrease in signal current was >5% after 4 injections. Since the electrostatic interaction attracts vasopressin to the BDD surface, the decrease in the current response was confirmed as being due to the strong adsorption of vasopressin at the BDD surface.

It has been reported that anodic oxidation at a high potential can recover the loss of signal resulting from adsorption at the BDD surface^32^. An experiment, where a potential is applied briefly to the electrode, was performed to optimize the time required to recover the signal current. Different durations between 10 and 60 s were investigated. The results showed that applying +3.0 V for 10 s was sufficient to recover the signal current. Hence, applying +3.0 V for 10 s was fixed as a pre-treatment.

**Figure 5:**
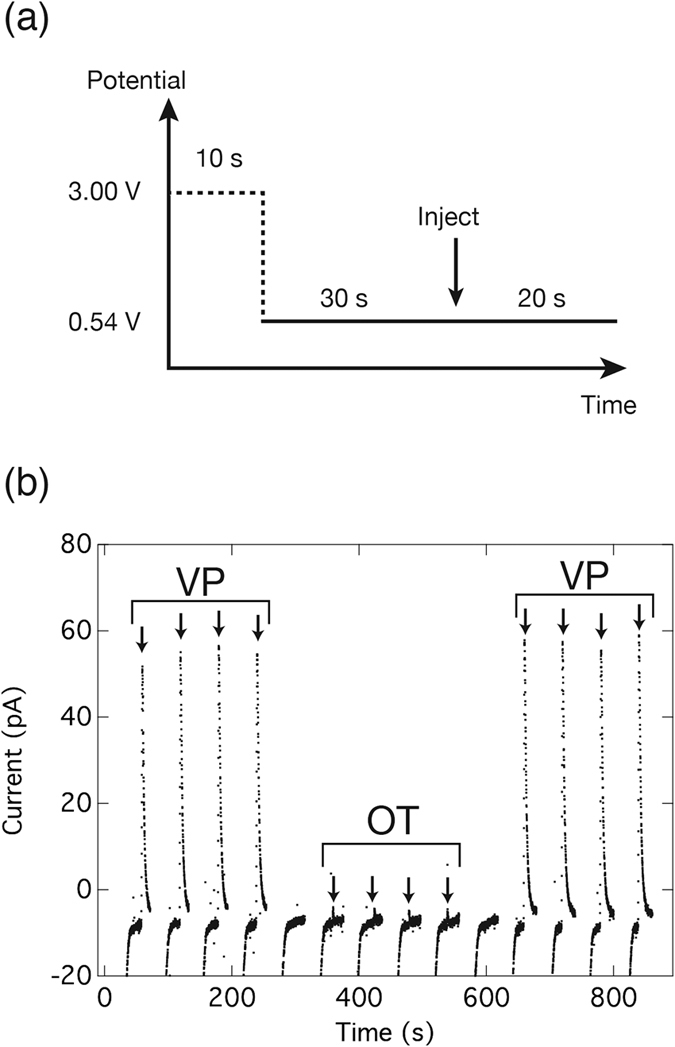
Selective detection of vasopressin and oxytocin on BDD microelectrode. (**a**) Potential step program used for chronoamperometry in the flow injection analysis system. (**b**) Consecutive signals in the selective detection of 10 μM vasopressin and 10 μM oxytocin using Tris (pH 7.4) as the buffer. Arrows indicate timings of sample injection. Note that there are two blank recordings between the vasopressin and oxytocin injections.

The measurement potential was then optimized at around +0.6 V, which is similar to the peak potential of vasopressin in the CVs shown in Fig. 3b. After the pre-treatment of +3.00 V for 10 s, the potential was stepped down to +0.6 V and the samples were injected 30 s after the potential step to stabilize the current. Including the 20 s after injection, the total time required for a single measurement was 60 s (Fig. 5a). This cycle was repeated four times for vasopressin, oxytocin, and back to vasopressin. Between the changes of sample from vasopressin and oxytocin, and *vice versa*, blank measurements without any sample injection were added. The measurements were performed with various applied potentials from +0.50 to +0.60. The results showed that at potentials of +0.54 V and below, the signal due to oxytocin can be minimized, and therefore, selective detection of vasopressin can be performed. Figure 5b shows consecutive injections of vasopressin and oxytocin in FIA at an applied potential of +0.54 V. A high signal for vasopressin can be observed, while the signal is almost zero for oxytocin, indicating that it is possible to use the method to distinguish between vasopressin and oxytocin.

Needless to say, if it is confirmed that it is only oxytocin that gets secreted by some trigger in the measurement, a simple measurement using a non-oxidized BDD microelectrode like Fig. 4 can be applied to detect oxytocin in a real biological sample. Further problems of the selectivity can be solved by the combination with measurements employing anodically oxidized BDD electrodes. Nevertheless, a measurement of the actual concentration of oxytocin *in situ* or *in vivo* has not yet been reported, and therefore, real-time detection of oxytocin using BDD electrodes would be a very useful device when treating patients with oxytocin and for the science related to this.

## Methods

### Preparation of electrodes

The BDD electrodes were prepared by growing polycrystalline BDD thin films on p-type single crystal silicon wafers (50 mm diameter, 0.75 mm thickness, 0.005–0.01 Ωcm resistivity) using a 2.45 GHz microwave plasma-assisted chemical vapor deposition system (Model AX5250M, ASTeX, Inc., Lowell, MA). Deposition was carried out at a plasma power of 5.0 kW for 8 h. The silicon wafers were prepared by abrading with diamond powder and then ultrasonicated in isopropyl alcohol. A mixture of acetone and trimethoxy borane was used as the carbon and boron sources, respectively, with a boron to carbon ratio of 1%. The deposition pressure was 100 Torr. Referring to the previous reports from our group^50^, the actual boron concentration in the BDD film can be presumed to be *ca*. 2.6 × 10^21^/cm^3^.

The BDD microelectrodes (Fig. 4a) were prepared by growing BDD thin films on tungsten wires. Initially, the 50 or 100 μm diameter tungsten wires were electrolytically polished in a solution of 1 M NaOH to achieve a conical shape (<10 μm in diameter at the tip). The BDD films were deposited on the tungsten wires using the same equipment used to deposit the BDD films on the silicon wafers, but a plasma power of 2.50 kW was applied for 3 h with a deposition pressure of 50 Torr. The BDD-coated tungsten wires were then insulated with only glass capillary and connected to tin-plated copper wire with silver paste. The size of the conducting tip of the electrode was <20 μm in diameter and 150–300 μm in length.

Characterization using scanning electron microscopy showed that the structure of both types of BDD electrodes was polycrystalline with a grain size in the range of 2–10 μm. Raman spectra of the boron-doped diamond were typical with a peak at ~1313 cm^−1^, which corresponds to sp^3^ diamond, and a couple of peaks at 470 and 1220 cm^−1^, which are related to the formation of disordered diamond and boron doping^51^,^52^. No peaks at ~1600 cm^−1^, due to non-diamond carbon impurities, were observed, indicating that the prepared BDD films were of a high quality. Because Raman spectra of both planar electrode and microelectrode were almost identical (see Supplementary Information Fig. S4), it can be expected that the boron concentration in microelectrodes were also the same value as that of planar BDD electrode.

Anodically-oxidized BDD electrodes were prepared by anodic oxidation of the as-deposited diamond on the electrodes at a potential of +3.0 V for 20 min in phosphate buffer solution (pH 7.4). Meanwhile, cathodic reduction at −3.0 V was applied to prepare cathodically-reduced BDD electrodes. Prior to use, the Pt and GC electrodes were polished using 0.5 μm alumina for 20 min, then ultrasonicated in pure water for 10 min.

### Electrochemical apparatus

Electrochemical measurements were conducted using a three-electrode system in a 0.8 mL single-compartment Teflon cell with BDD, Pt or GC as the working electrode, while Pt wire and Ag/AgCl (KCl saturated) were used as the counter and reference electrodes, respectively. The BDD (Pt and GC) electrode was fixed with an O-ring (electrode area: 0.36 cm^2^) and connected to a potentiostat through a copper plate placed under the BDD electrode. Data was recorded by an Autolab potentiostat (PGSTAT101, Metrohm Autolab B.V.) using NOVA software (version 1.11). For the flow injection analysis, a BDD microelectrode was affixed to the output of flow injection apparatus consisting of a six-port HPLC loop injector (Rheodyne model 9725i valve) and a HPLC pump (model PU 712, GL sciences Inc., Japan). The construction of this flow system is based on one described in a published paper^53^. The tip of the microelectrode was inserted 1 mm into the 0.8 mm diameter outlet tube. The flow rate was 1 mL/min and the volume of sample injected was 20 μL.

### Chemicals

Oxytocin (Cys-Tyr-Ile-Gln-Asn-Cys-Pro-Leu-Gly-NH_2_ (disulfide bond between Cys1-Cys6)) and [Arg^8^]-vasopressin (Cys-Tyr-Phe-Gln-Asn-Cys-Pro-Arg-Gly-NH_2_ (disulfide bond between Cys1-Cys6)) were purchased from the PEPTIDE INSTITUTE, INC., Japan, and were used without any further purification. A solution of 0.1 M phosphate buffer solution (PBS) or Tris buffer (15 mM H_2_NC(CH_2_)(OH)_3_·HCl, 140 mM NaCl, 3.25 mM KCl, 1.2 mM CaCl_2_, 1.25 mM NaH_2_PO_4_·H_2_O, 1.2 mM MgCl_2_ and 2.0 mM Na_2_SO_4_) were used as the electrolyte after adjusted to pH 7.4. All the solutions were prepared with pure water supplied from DIRECT-Q 3 UV (Merck Millipore Corporation) with a specific resistivity of 18.2 MΩ cm.

## Additional Information

**How to cite this article**: Asai, K. *et al*. Continuous and selective measurement of oxytocin and vasopressin using boron-doped diamond electrodes. *Sci. Rep.* **6**, 32429; doi: 10.1038/srep32429 (2016).

## Supplementary Material

Supplementary Information

## Figures and Tables

**Table 1:** Acid dissociation constant (p*K*_a_) of the phenolic group of oxytocin, vasopressin, and tyrosine and isoelectric point (pI) of the three molecules^48^,^49^ and some related amino acids.

	p*K*_a_ of the OH group	pI
Tyrosine	10.0	5.7
Oxytocin	10.1	7.7
Vasopressin	9.8	10.9
Isoleucine	—	5.9
Leucine	—	6.0
Phenylalanine	—	5.5
Arginine	—	11.2
