# Supplementary material for: Continuous and selective measurement of oxytocin and vasopressin using boron-doped diamond electrodes
Source: Sci Rep. 2016 Sep 7;6:32429. doi: 10.1038/srep32429 (PMC5013270; doi:10.1038/srep32429)
Supplement: Supplementary Information [file srep32429-s1.doc]

**Supplementary Information:**

# **Continuous and selective electrochemical measurement of oxytocin and vasopressin using boron-doped diamond electrodes**

Kai Asai^1^, Tribidasari A. Ivandini^2^, Yasuaki Einaga*^1,3^

^1^Department of Chemistry, Faculty of Science and Technology, Keio University, 3-14-1 Hiyoshi, Yokohama 223-8522, Japan

^2^Department of Chemistry, Faculty of Mathematics and Science, University of Indonesia, Kampus UI Depok, Jakarta 116424, Indonesia

^3^JST-ACCEL, 3-14-1 Hiyoshi, Yokohama 223-8522, Japan

E-mail: einaga@chem.keio.ac.jp

**Figure S1 | Analysis of reduction signal of oxytocin.** Cyclic voltammograms of 0.1 mM oxytocin in PBS (0.1 M, pH 7.4) using as-deposited BDD electrodes. 1^st^ scan was 0 →−0.6 → 0 V, 2^nd^ and 3^rd^ scans were 0 → −0.6 → +1.2 → 0 V. Note that the reduction signal at −0.4 V did not appear without the oxidation reaction (see the 2^nd^ scan). The other conditions are similar to those in Figure 1(a).

**Figure S2 | Cyclic voltammograms of oxytocin and cystine.** Cyclic voltammograms of 0.1 mM oxytocin (OT) and cystine (CySSyC) in PBS (0.1 M, pH 7.4) using as-deposited BDD electrodes. The other conditions are similar to those in Figure 1(a).


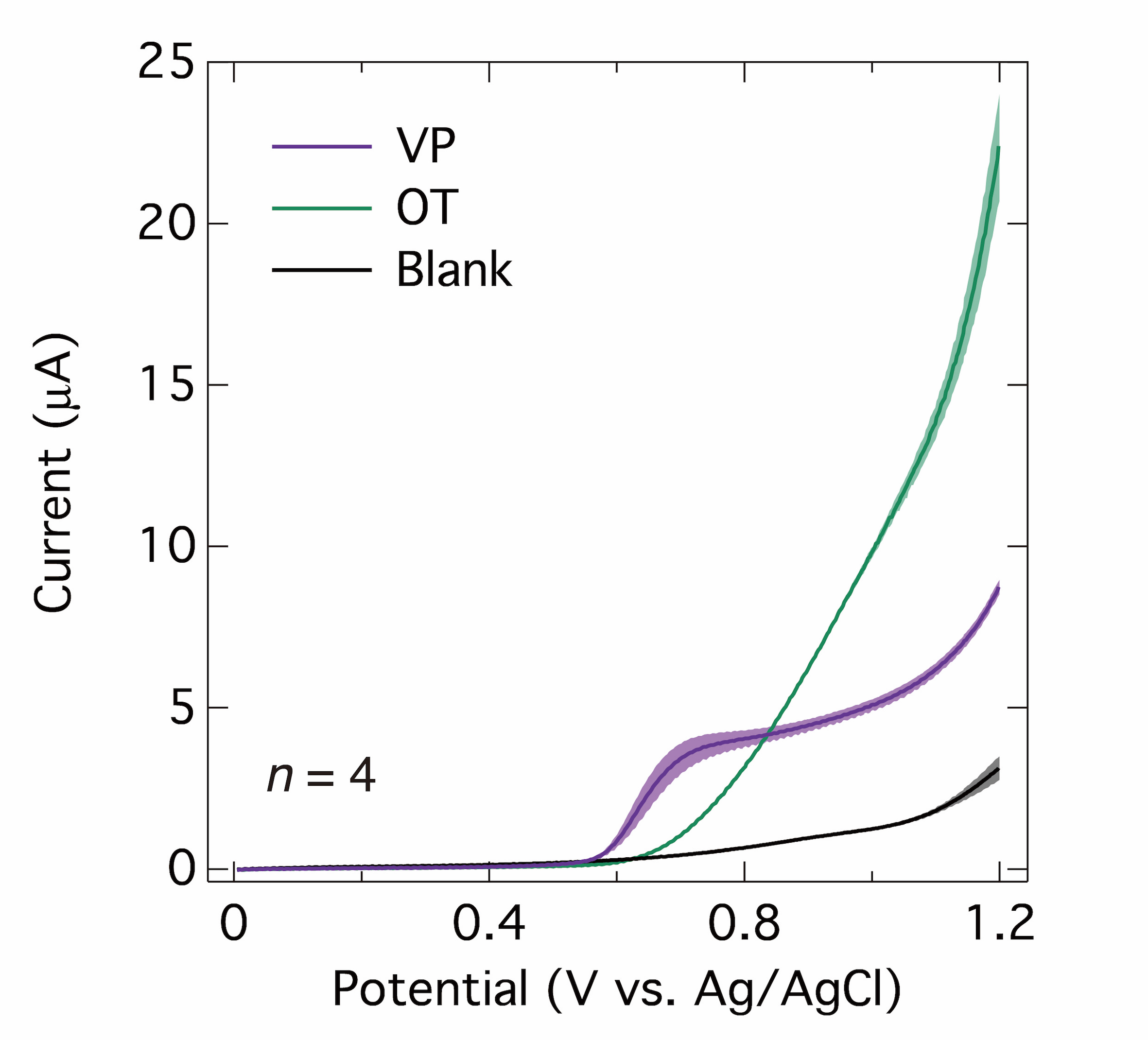


**Figure S3 | Linear sweep voltammograms of oxytocin and vasopressin on AO-BDD in Tris buffer.** Cyclic voltammograms of Tris buffer (pH 7.4) using an anodically oxidized BDD microelectrode in comparison with voltammograms in the presence of 0.1 mM oxytocin (OT) and vasopressin (VP). The other conditions are similar to those in Figure 1(a).

**Figure S4 | Raman spectra of BDDs.** (a) Typical Raman spectrum of planar BDD thin film. (b) Typical Raman spectrum of BDD thin film on microelectrode.

(a)

(b)
